# Three-dimensional imaging for the localization of related anatomical structures during surgery on the internal auditory canal

**DOI:** 10.1186/s12893-022-01527-w

**Published:** 2022-03-02

**Authors:** Ying Guo, Mengxing Li, Kailiang Cheng, Youqiong Li, Qingjie Ma

**Affiliations:** 1 Department of Nephrology, China-Japan Union Hospital of Jilin University, Changchun, 130033 China; 2 Department of Human Anatomy, Norman Bethune College of Medicine, Jilin University, Changchun, 130021 China; 3 Departments of Nuclear Medicine, China-Japan Union Hospital of Jilin University, No. 126, Xiantai St, Changchun, 130033 Jilin China

**Keywords:** Arcuate eminence, Superior semicircular canal, Internal auditory canal, Fisch’s method, CT three-dimensional imaging

## Abstract

**Background:**

The Fisch infra-temporal fossa approach (Fisch’s method), first proposed in 1970, is commonly used during internal auditory canal (IAC) surgery with an approach that advances through the middle cranial fossa. This study was designed to address the technical difficulties encountered in recognizing and localizing the arcuate eminence with respect to the superior semicircular canal (SSC).

**Methods:**

Forty men and 40 women (18–57 years of age) without space-occupying lesions in the petrous part of the temporal bone were selected for the study. In total, 160 samples were obtained from both sides of the temporal bone. The temporal bone in these 160 samples was scanned using computed tomography, and a three-dimensional coordinate system was established to measure the three-dimensional coordinate values of structures adjacent to the arcuate eminence, the SSC, and the IAC.

**Results:**

The results showed that the shape of the arcuate eminence is highly variable. Approximately 23.12% of samples had no obvious arcuate eminence, which prevented the use of Fisch’s method to localize the SSC. The arcuate eminence was difficult to identify in 37 samples.

**Conclusions:**

Analysis samples showed that the SSC was located in a fan ring centered at the midpoint of the upper edge of the petrous portion of the temporal bone. The arcuate eminence did not correspond directly with the SSC, as the former was located posterolateral to the latter in 85.83% of samples. The angle between the SSC and the IAC ranged from 0° to 60° degrees, as reported previously by Fisch. However, the angle typically ranged from 10–30° in our study.

## Background

Experts around the world have explored different surgical approaches to remove acoustic neuromas, preserving facial nerve function. These methods include the retrosigmoid sinus approach, the translabyrinthine approach, and the middle cranial fossa approach [1]. The latter approach fully exposes the bottom and lateral parts of the internal auditory canal (IAC) during surgery, enabling visualization of the facial and auditory nerves during the removal of acoustic neuromas and preserving facial nerve function. In 1963, William House performed surgery in the IAC via the middle cranial fossa [2]. In this study, we built upon Doctor HousE′s proposed method for localization of the IAC. Furthermore, with the development of neuromicrosurgery, it has become possible to operate with precision over the structures surrounding the IAC using an approach through the middle cranial fossa. Localizing the IAC and thus avoiding damage to adjacent structures is of paramount importance for the feasibility of said surgery. Fisch’s method is one of many methods used for localization of the IAC via the middle cranial approach. This technique, using the arcuate eminence as a landmark, helps to prevent damage to the facial nerve. However, clinical practice has shown that it is difficult to identify the arcuate eminence and to characterize the positional relationship between the arcuate eminence and the superior semicircular canal (SSC), which may lead to intraoperative injury of the SSC and postoperative vertigo [3].

In this study, a three-dimensional coordinate system was established after images were obtained with computed tomography (CT). Reference points for the arcuate eminence, the SSC, and the IAC were selected, and the corresponding coordinate values were measured. These coordinate values were used to analyze the shape and mutual positional relationship of each structure. This approach improves upon the Fisch method to shorten the surgery time, reduce the risk of complications, and improve the safety.

## Methods

### Data collection

Forty men and 40 women were randomly selected, with a total of 160 samples obtained from the left and right sides. The individuals ranged in age from 18 to 57 years, with an average age of 46 years. Two experienced radiologists confirmed that there were no space-occupying lesions in the petrous portion of the temporal bone.

This study was approved by the Ethics Committee of the China-Japan Union Hospital of Jilin University. All procedures performed in studies involving human participants were in accordance with the ethical standards of the institutional and national research committee and with the 1964 Helsinki Declaration and its later amendments or comparable ethical standards. Written informed consent was obtained from all individual participants included in this study.

### CT scanning of temporal bone

The scanning instrument was a 64-slice spiral CT from the China-Japan Union Hospital of Jilin University (Discovery CT 750HD; GE Healthcare, Milwaukee, USA). With the orbitomeatal (OM) line as the baseline, the temporal bone was scanned in the axial position (layer thickness, 0.625 mm). The bone window image was measured.

### Three-dimensional reconstruction technology

The CT data were recorded as a DICOM file and transmitted to a workstation (GE AW 4.6) for reconstruction.

### Establishing a three-dimensional (3D) coordinate system

**Fig. 1:**
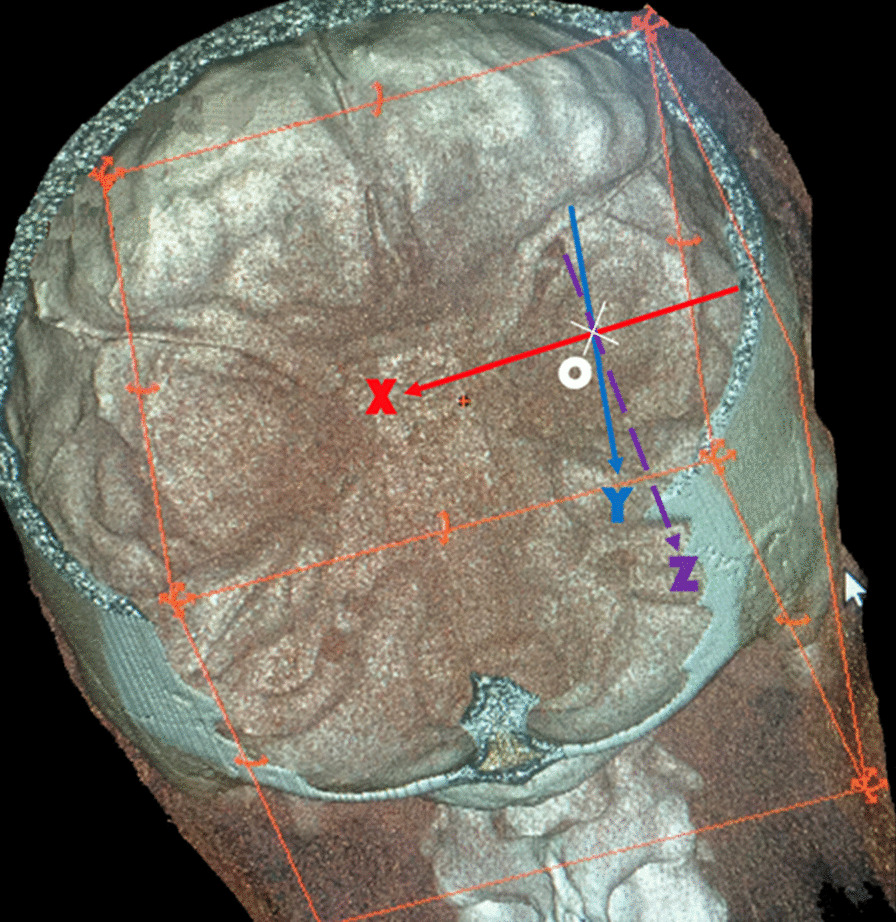
Establishing a three-dimensional coordinate system. O: the highest point of the arcuate eminence, X-axis: parallel to the coronal plane, Y-axis: parallel to the sagittal plane, Z-axis: vertical to the horizontal plane (the Frankfurt plane)

The highest point of the arcuate eminence (point O) was set as the origin. The x-axis was designed to be parallel to the coronal plane, the y-axis was designed to be parallel to the sagittal plane, and the z-axis was designed to be vertical to the horizontal plane (the Frankfurt plane). The positive direction was toward the left, back, and feet for the x-, y-, and z-axis, respectively (Fig. 1).

The authors used a positive direction toward the left, unlike in the standard stereotactic system, purely for the purpose of simplicity. As the IAC is to the left of the (right) arcuate eminence and the latter is used here as the “origin (O)”, taking the right temporal bone and right cranial cavity as references, all the vectors leading away from origin (O) to the structures of interest were denoted as positive.

### Selection of anatomical reference points

**Fig. 2:**
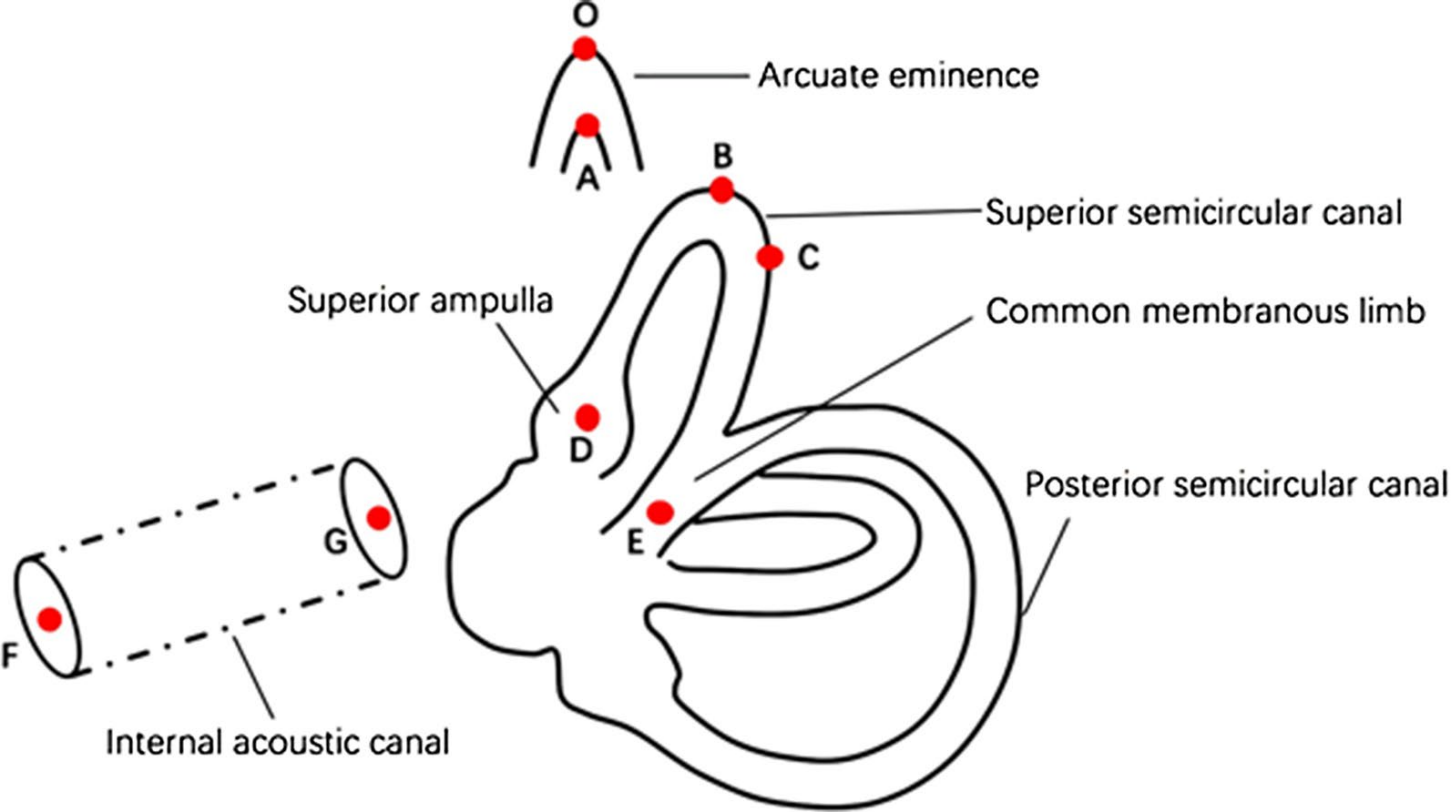
Selection of anatomical reference points. **A** The lower margin of the bony arcuate eminence, **B** the highest point of the SSC, **C** the lateral point of the SSC, **D** the ampulla limb point of the SSC, **E** the common limb point of the SSC, **F** the internal auditory opening, **G** the center of the bottom of the internal auditory canal

In the established 3D coordinate system, reference points were selected to localize the arcuate eminence, the SSC, and the IAC. The lower margin of the bony arcuate eminence, the highest point of the SSC, the ampulla limb point of the SSC (center of the ampulla limb section), and the common limb point of the SSC (center of the common limb section) were selected horizontally as points A, B, D, and E, respectively. The lateral point of the SSC (point C) was selected in the sagittal plane. Point F and point G were located in the sagittal and coronal planes, respectively. Point F was at the internal auditory opening (center of the internal auditory meatus section), and point G was the center of the bottom of the IAC (Fig. 2).

### Parameter measurements

Points A–G were projected to the x-, y-, and z-axes, respectively. Distances between the projected points and the origin O were measured in mm. These distances were taken as the coordinates of points A–G and recorded.

Because the computations were performed using 3D simulation, and also since surgery (open, endoscopic, or stereotactic) was performed with image guidance, the distances were all measured using the Euclidean Distance Transformation (EDT) technique [4]. All the relationships were in 3D, not 2D, as EDT was applied.

### Data processing and statistical analysis

Statistical analysis was performed using the SPSS 28.0 statistical software package. The Shapiro Wilk test indicated the measured data conformed to the normal distribution. The quantitative data were expressed as the mean ± standard deviation. The differences between groups of continuous data were identified with the Student’s t-test. A value of P < 0.05 was considered statistically significant. Origin O and point A were used to describe the shape and location of the arcuate eminence and to measure its thickness. Points B–E were used to determine the position of the SSC. The positional relationship between the arcuate eminence and the SSC was also analyzed. Points B, D, and E were used to map out the direction of the SSC, while points F–G were used parallel to the direction of the IAC to determine the angle between structures.

## Results

### Measuring shape and thickness of the arcuate eminence

**Fig. 3:**
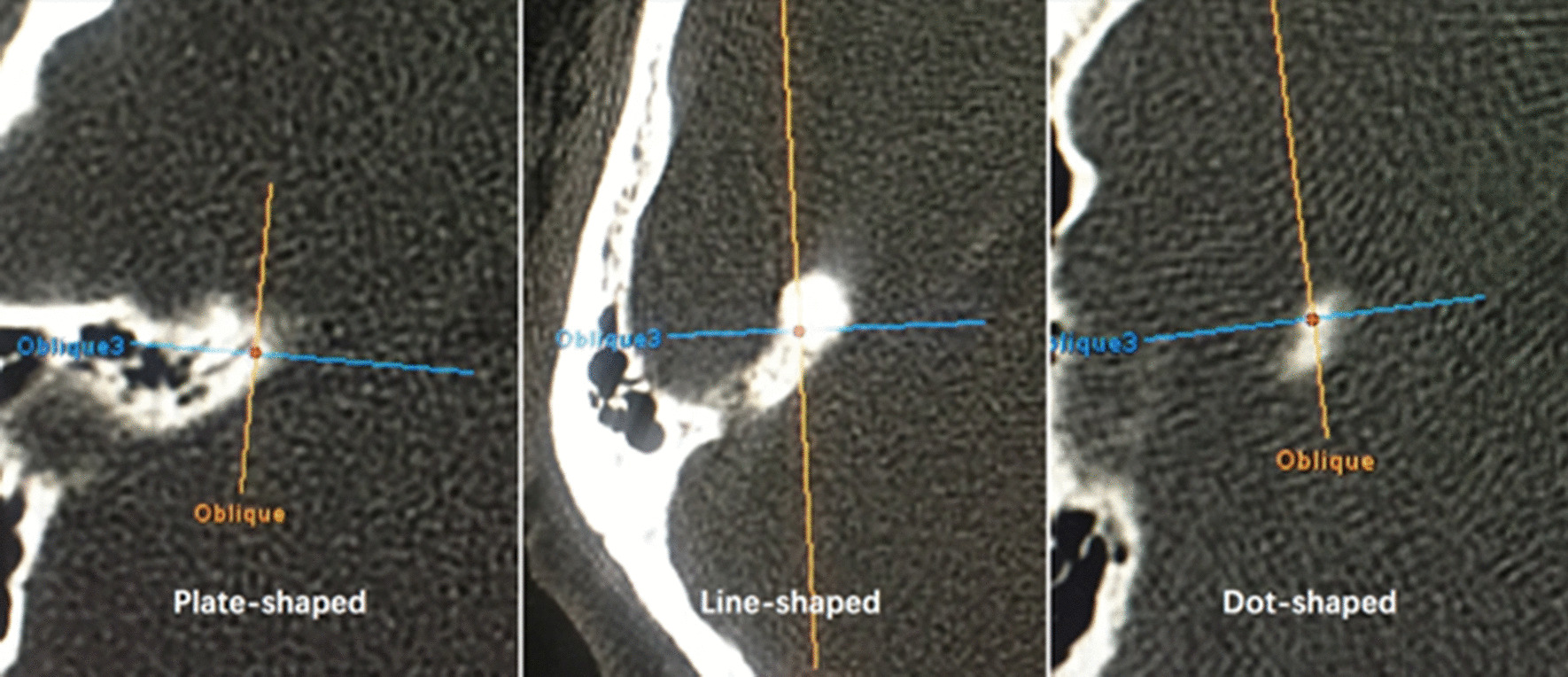
Morphology of arcuate eminence

We found that the shape of the arcuate eminence could be divided into three types: planar, linear, or point-like. Since only the “point-like” pattern of the arcuate eminence was chosen for sampling, and in order to distribute the data equitably and optimally, a range of EDTs was used to denote the “bulk and form” of the arcuate eminence, and all the data samples could be fitted into this range. The arcuate eminence was classified as follows: planar=plate-shaped (difficult to identify); linear=line-shaped; and point-like=dot-shaped (Fig. 3).

The planar and linear types were excluded from the sampling, as their exact measurements could not be substantiated.

The proportion of arcuate eminence samples among the total collection of 160 samples exhibiting a given shape was calculated (Table 1), and 120 samples (male, n = 60; female, n = 60) with arcuate eminences characterized by point-like protrusion were selected for subsequent analysis. The thickness of the arcuate eminence was measured, and the results were expressed as the mean ± standard deviation. The mean thickness of the left arcuate eminence was 1.82 ± 0.45 mm (range 0.9–3.2), and that of the right arcuate eminence was 1.77 ± 0.37 mm (range 0.9–3.1). Overall mean thickness was 1.69 ± 0.15 mm; P = 0.289 (no statistical significance).

**Table 1:** Statistical analysis of the shape of the arcuate eminence (number of cases)

Item	Total	Planar	Linear	Point–like
Total	160	37 (23.12%)	3 (1.88%)	120 (75.00%)
Male	80	19 (23.75%)	1 (1.25%)	60 (75.00%)
Female	80	18 (22.50%)	2 (2.50%)	60 (75.00%)

Planar plate-shaped (difficult to identify)

Linear line-shaped

Point-like dot-shaped

### Differential detection on the left vs. right sides of the sample

We selected 120 samples (male, n = 60; female, n = 60) with arcuate eminence characterized by point-like protrusions from among the 160 samples initially included in the study. The skull is a bilaterally symmetrical structure; hence the point opposite “x” should be compared to “x” on the right side. All other values in Table 2 are raw data (Table 2).

**Table 2:** Significance of differences among coordinates A–E (±s)

(n = 120)	x			y			z		
	L(–x)	R	P	L	R	P	L	R	P
A	0	0	Null	0	0	null	1.82 ± 0.45	1.77 ± 0.37	0.293
B	3.45 ± 2.18	3.70 ± 2.91	0.799	–3.39 ± 2.18	–3.06 ± 2.16	0.335	2.54 ± 0.99	2.67 ± 1.05	0.129
C	2.51 ± 2.67	2.24 ± 2.86	0.582	–6.56 ± 2.56	–6.69 ± 2.80	0.478	7.66 ± 1.40	7.59 ± 1.39	0.682
D	3.78 ± 1.84	3.80 ± 2.89	0.950	–5.91 ± 2.37	–5.69 ± 2.59	0.54	8.56 ± 1.61	8.38 ± 1.29	0.848
E	6.08 ± 2.26	5.96 ± 2.81	0.768	–1.43 ± 2.27	–1.22 ± 2.45	0.565	8.25 ± 1.38	8.27 ± 1.27	0.916
F	16.73 ± 2.74	16.51 ± 3.49	0.792	–1.53 ± 3.05	–1.90 ± 3.10	0.351	9.58 ± 1.90	10.09 ± 1.70	0.290
G	8.23 ± 2.26	7.94 ± 3.37	0.562	–7.06 ± 2.45	–6.75 ± 3.18	0.478	8.44 ± 1.40	8.45 ± 1.39	0.914

L left side

R right side

A the lower margin of the bony arcuate eminence

B the highest point of the SSC

C the lateral point of the SSC

D the ampulla limb point of the SSC (center of the ampulla limb section)

E the common limb point of the SSC (center of the common limb section)

F the internal auditory opening (center of the internal auditory meatus section)

G the center of the bottom of the internal auditory canal

### Assessment of the positional relationship between the arcuate eminence and the SSC

**Fig. 4:**
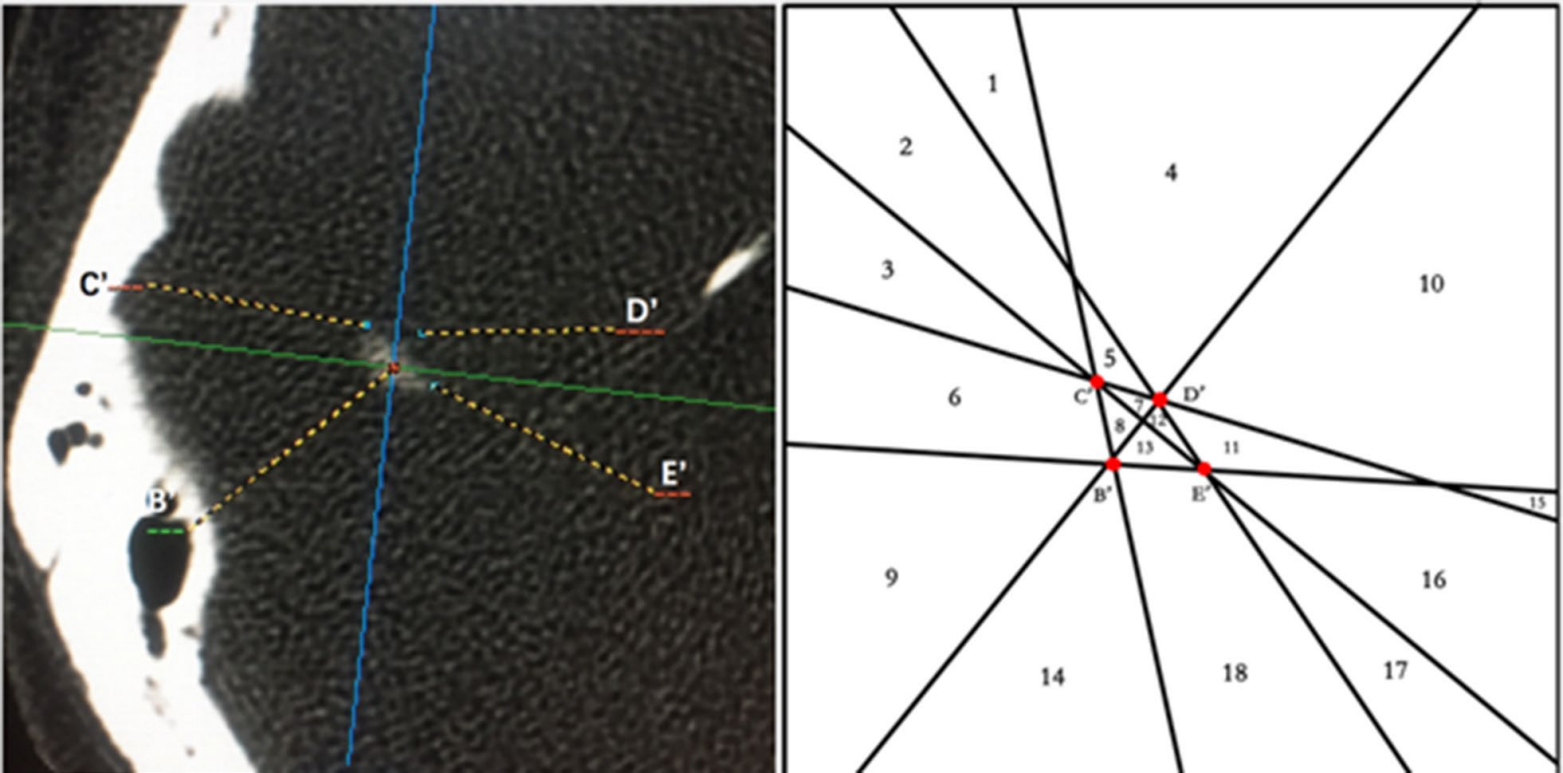
Points **B**′–**E**′: the projection of the highest point of the SSC, **C**’: the projection of the lateral point of the SSC, **E**’ and the 18 areas cut by their links. **D**’: the projection of the ampulla limb point of the SSC, **E**’: the projection of the common limb point of the SSC

From among 160 samples, 120 samples (male, n = 60; female, n = 60) with arcuate eminences characterized by point-like protrusion were selected for assessment of the positional relationship between the arcuate eminence and the SSC. In all samples, when points B–E were projected onto the horizontal plane, the four points formed a quadrilateral (B′C′D′E′). The connections among B′C′, C′D′, D′E′, E′B′, B′D′, and C′E′ divided the plane into 18 areas (Fig. 4). Calculations were performed as follows:

Calculate the value of ${\text{Location}}_{{{\text{OE}{^{\prime}}} \sim {\text{C}{^{\prime}D{^{\prime}}}}}} = \left[ {\left( {\left( {{\text{x}}_{{\text{O}}} - {\text{x}}_{{\text{C}}} } \right)\left( {{\text{y}}_{{\text{D}}} - {\text{y}}_{{\text{C}}} } \right){\text{ }} - \left( {{\text{y}}_{{\text{O}}} - {\text{y}}_{{\text{C}}} } \right)\left( {{\text{x}}_{{\text{D}}} - {\text{x}}_{{{\text{C}})}} } \right)} \right)} \right]\left[ {\left( {{\text{x}}_{{\text{E}}} - {\text{x}}_{{\text{C}}} } \right)\left( {{\text{y}}_{{\text{D}}} - {\text{y}}_{{\text{C}}} } \right){\text{ }} - {\text{ }}\left( {{\text{y}}_{{\text{E}}} - {\text{y}}_{{\text{C}}} } \right)\left( {{\text{x}}_{{\text{D}}} - {\text{x}}_{{\text{C}}} } \right)} \right] .$

If ${\text{Location}}_{{{\text{OE}{^{\prime}}} \sim {\text{C}{^{\prime}D{^{\prime}}}}}}$> 0, then O and E′ are on the same side of line C′D′.

If ${\text{Location}}_{{{\text{OE}{^{\prime}}} \sim {\text{C}{^{\prime}D{^{\prime}}}}}}$ < 0, then O and E′ are on opposite sides of line C′D′.

If ${\text{Location}}_{{{\text{OE}{^{\prime}}} \sim {\text{C}{^{\prime}D{^{\prime}}}}}}$= 0, then O and E′ are both on line C′D′.

**Fig. 5:**
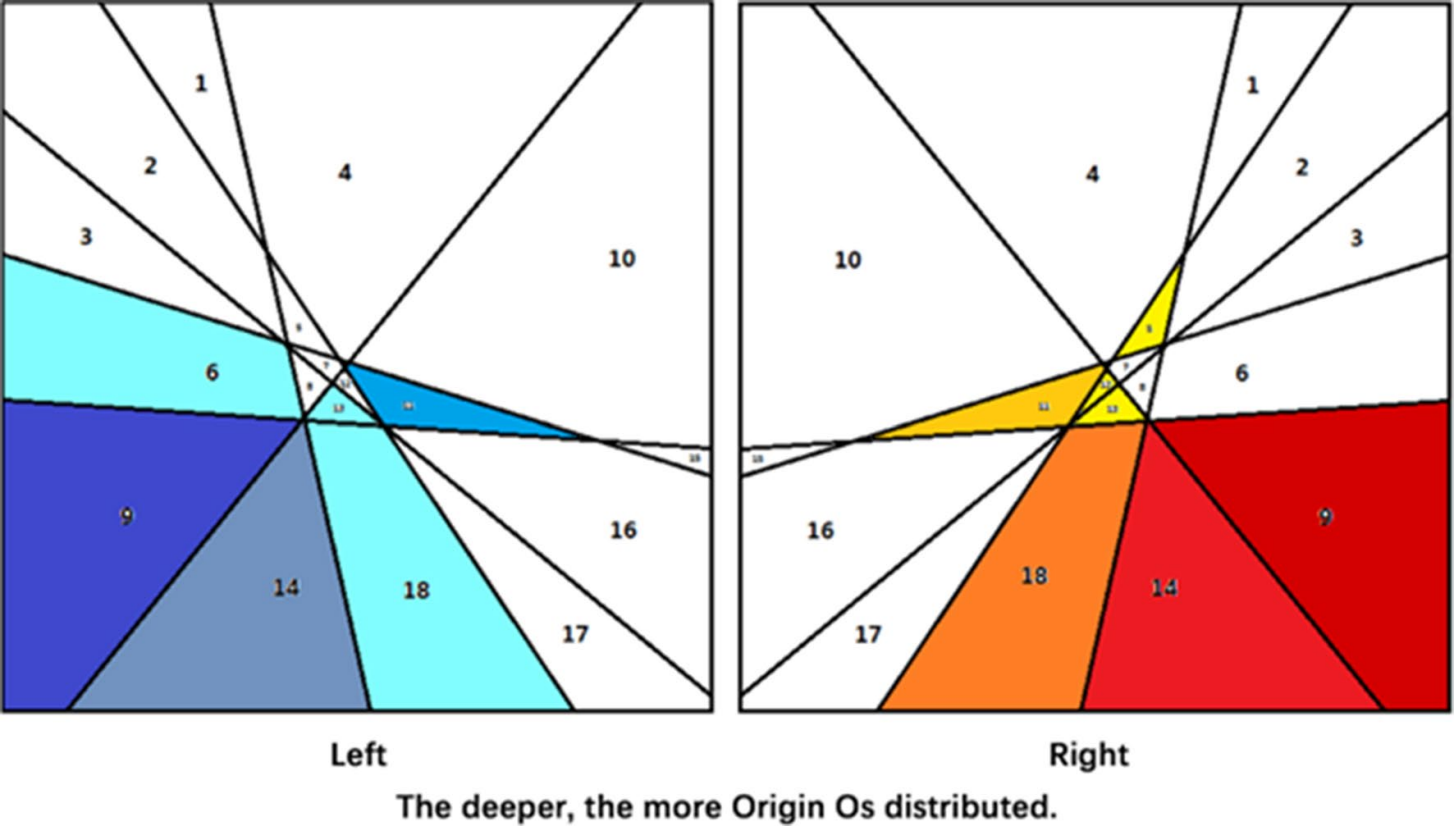
Distribution of origin O in Area_1_~Area_18_

Calculate the value of ${\text{Location}}_{{{\text{OC}{^{\prime}}} \sim {\text{D}{^{\prime}E{^{\prime}}}}}}$, ${\text{Location}}_{{{\text{OD}{^{\prime}}} \sim {\text{C}{^{\prime}E{^{\prime}}}}}}$, ${\text{Location}}_{{{\text{OB}{^{\prime}}} \sim {\text{C}{^{\prime}E{^{\prime}}}}}}$, ${\text{Location}}_{{{\text{OC}{^{\prime}}} \sim {\text{B}{^{\prime}E{^{\prime}}}}}}$, ${\text{Location}}_{{{\text{OE}{^{\prime}}} \sim {\text{B}{^{\prime}C{^{\prime}}}}}}$,${\text{Location}}_{{{\text{OE}{^{\prime}}} \sim {\text{C}{^{\prime}D{^{\prime}}}}}}$ , and ${\text{Location}}_{{{\text{OC}{^{\prime}}} \sim {\text{B}{^{\prime}D{^{\prime}}}}}}$L to quantify the 18 areas (Corresponding values for Area_1_–Area_18_ are presented in Table 3). Substitute the sample data [(x_O_,y_O_) and (x_B_,y_B_)–(x_E_, y_E_)] into the formula. The distribution of the highest point of the arcuate eminence (point O) from Area_1_ to Area_18_ was analyzed for each sample (Table 4) (Fig. 5).

**Table 3:** Location values for each area

Area	OE′~C′D′	OD′~C′E′	OC′~D′E′	OE′~B′C′	OC′~B′E′	OB′~C′E′	OC′~B′D′
1	–	+	–	–	+	–	+
2	–	+	+	–	+	–	+
3	–	–	+	–	+	+	+
4	–	+	–	+	+	–	+
5	–	+	+	+	+	–	+
6	+	–	+	–	+	+	+
7	+	+	+	+	+	–	+
8	+	–	+	+	+	+	+
9	+	–	+	–	–	+	+
10	–	+	–	+	+	–	–
11	+	+	–	+	+	–	–
12	+	+	+	+	+	–	–
13	+	–	+	+	+	+	–
14	+	–	+	–	–	+	–
15	–	+	–	+	–	–	–
16	+	+	–	+	–	–	–
17	+	–	–	+	–	+	–
18	+	–	+	+	–	+	–

**Table 4:** Distribution of origin O in Area_1_–Area_18_ (number of cases)

Area	L	R	Total	Percentage (%)
All	60	60	120	100
1	0	0	0	0.00
2	0	0	0	0.00
3	0	0	0	0.00
4	0	0	0	0.00
5	0	1	1	0.83
6	1	0	1	0.83
7	0	0	0	0.00
8	0	0	0	0.00
9	47	41	88	73.33
10	0	0	0	0.00
11	3	3	6	5.00
12	0	1	1	0.83
13	1	2	3	2.50
14	6	9	15	12.50
15	0	0	0	0.00
16	0	0	0	0.00
17	0	0	0	0.00
18	2	3	5	4.17

### Angle formed by the SSC and the internal auditory canal

From among 160 samples, 120 samples (male, n = 60; female, n = 60) with arcuate eminences characterized by point-like protrusions were selected for analysis of the angle formed by the SSC and the IAC. The vector $\overrightarrow {{{\text{FG}}}}$ was used to fit the IAC. After taking the midpoint M of points D and E, the vector $\overrightarrow {{{\text{MB}}}}$ was used to fit the SSC. The angle between $\overrightarrow {{{\text{FG}}}}$ and $\overrightarrow {{{\text{MB}}}}$ was designated as θ, representing the angle formed by the SSC and the IAC (Table 5).

**Table 5:** Angle formed by internal acoustic canal and superior semicircular canal (number of cases)

*cosθmax*	1.00	0.99	0.97	0.94	0.91	0.87	0.86	0.77	0.71	0.64	0.57	0.50
*cosθmin*	0.99	0.97	0.94	0.91	0.87	0.86	0.77	0.71	0.64	0.57	0.50	0.00
θ	0–10	10–15	15–20	20–25	25–30	30–35	35–40	40–45	45–50	50–55	55–60	60–90
L	0	13	15	14	13	1	1	2	0	0	0	1
R	1	11	17	14	10	3	1	0	1	1	0	1
Total	1	24	32	28	23	4	2	2	1	1	0	2

L left side

R right side

## Discussion

Historically, the IAC has been accessed for the removal of cerebellopontine angle tumors, including acoustic neuromas, using a number of different surgical approaches such as the middle fossa, retrolabyrinthine, translabyrinthine, supralabyrinthine, subtemporal [5], and retrosigmoid [6]. These can be a combination of open and endoscopic procedures, or even purely endoscopic [7]. Additionally, image guidance and facial nerve monitoring have helped refine both the technical efficacy and safety of these methods. Though the middle cranial fossa approach remains one of the most popular methods for access, and is the one being discussed in this study, the relationship between the important landmarks is a valid consideration for any of the above approaches [8].

Shiobara in 2008 and Ahmad in 2012 reported that the rate of complete resection and recurrence when performing surgery for the removal of acoustic neuroma via the middle cranial fossa approach was 94.1% and 1.8%, respectively [9, 10]. They demonstrated that the middle cranial fossa approach had advantages for the resection of tumors in the IAC. However, the IAC is deep and concealed, and the surrounding anatomical structures are complex. As a result, the rates of complete tumor resection and likelihood that facial nerve function will be preserved after surgery on the internal ear canal remain unsatisfactory.

Microsurgeons have proposed several methods for anatomical localization, including those proposed by House, Fisch, Garcia-Ibanez, and Catalano. Each technique is designed to increase the likelihood that the surgeon will accurately enter the IAC without endangering adjacent anatomical structures. HousE′s method localizes the IAC using the nervi petrosus superficialis major and the facial nerve tube hiatus as landmarks [2]. Fisch’s method determines the position of the SSC in relation to the arcuate eminence, then locates the IAC in relation to the SSC [11]. Garcia-Ibanez proposed that the facial nerve canal hiatus and arcuate eminence be used as landmarks for joint localization [12]. Catalano proposed use of the roots of the zygoma and the head of the malleus in the middle ear for joint localization [13].

Fisch’s method was first introduced in 1970. The primary advantage of this approach is that the facial nerve is seldom injured; however, the limitation of this approach is the high risk for intra-operative injury of the SSC, and consequently, postoperative vertigo. Long-term research and practice have shown that the reason for this high risk of injury is that the SSC is quite complex. On the other hand, the morphology of the arcuate eminence and its positional relationship to the SSC remain controversial. The method of IAC positioning proposed by scholars is intended to compensate for the shortcomings of Fisch’s method in order to increase the rate of success of the surgery and thus avoid complications.

In this study, the results obtained with CT scanning technology and a 3D coordinate system were used to characterize temporal bone anatomy. The results allowed clinicians to identify the arcuate eminence and further clarify the positional relationship between the arcuate eminence and the SSC.

### Measuring the shape and thickness of the arcuate eminence

**Fig. 6:**
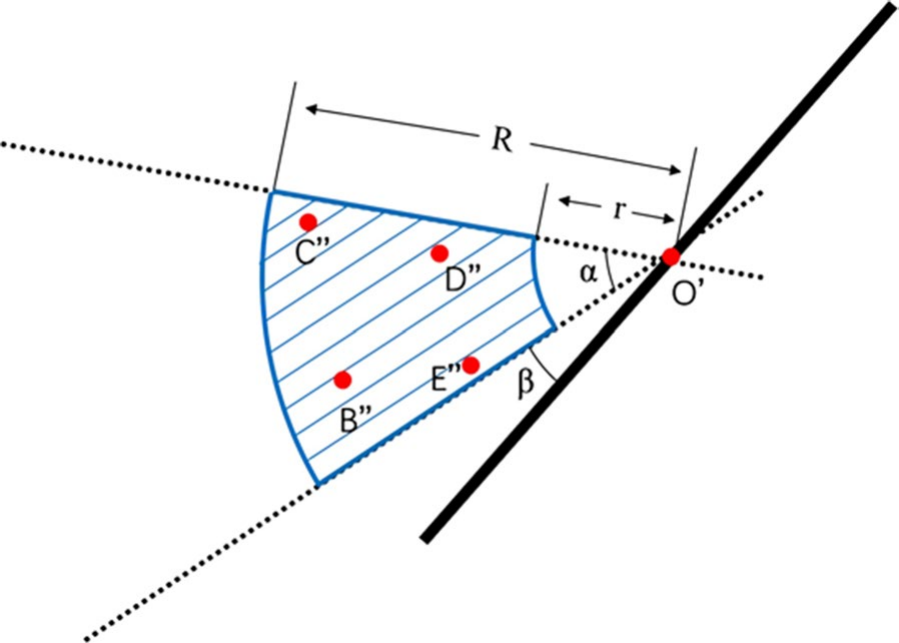
Relationships between O’ and B′’~E′’. O’: the midpoint of the upper margin of the temporal bone, B′’~E′’: the projections of B ~ E in cases in which the arcuate eminence was inconspicuous

In 2003, Faure studied 100 cases with high-resolution CT of the temporal bone. In 15% of the cases, it was difficult to accurately identify the arcuate eminence [14]. In 2007, Seo et al. studied 52 high-resolution CT images of temporal bones in 26 patients. The results of the study showed that the arcuate eminence could not easily be identified in nine cases (34.6%) [15]. The results of statistical analysis showed that the shape of the arcuate eminence was indefinite, and the structure was difficult to identify in 23.12% of cases. In the remaining cases, the arcuate eminence had a shape characterized by linear or point-like protrusions. Through 3D coordinate measurement, the average thickness of the arcuate eminence was determined to be 1.69 ± 0.15 mm (range 0.9-3.2), with no significant laterality effect. Thirty-seven cases in which the arcuate eminence was inconspicuous were analyzed. The origin was moved to the midpoint of the upper margin of the temporal bone and labeled as O’. The coordinates of points B–E were measured, and the points were then projected onto the horizontal plane to obtain B′’–E′’. It was found that points B′’–E′’ were distributed in a ring centered on O’. The parameters of the ring were as follows: R = 18.07 mm, r = 6.80 mm, α = 50 degrees, and β = 10 degrees (Fig. 6).

### The positional relationship between the arcuate eminence and the SSC

Although clarifying the relationship between the arcuate eminence and the SSC is the key to Fisch’s method, this point has attracted some controversy. Faure found that 37% of samples had a consistent positional relationship between the arcuate eminence and the SSC, but the former was not a good landmark for identifying the latter in 48% of samples [14]. Seo et al. showed that the arcuate eminence corresponded exactly with the SSC in only 2/52 petrous bones, corresponded well in 7/52, was lateral to the SSC in 25/52 cases, was medial to the SSC in 6/52 cases, intersected the SSC in 3/52 cases, and was indiscernible in 9/52 cases [15]. Guo et al. conducted a 3D imaging study on the anatomical markers used for acoustic neuroma surgery and concluded that the arcuate eminence was not the most accurate landmark for judging the SSC [16]. In our study, the lines connecting the reference points of the semicircular canal cut the plate into 18 areas. We found that the arcuate eminence did not correspond to the SSC completely (Fig. 5).

Among 120 samples included in the study, the arcuate eminence was located posterolateral to the SSC in 103 samples (Area_9_+Area_14_), accounting for 85.83% of cases. The posterior and anterior parts of the SSC were the blank areas of the arcuate eminence distribution, meaning that the arcuate eminence was not located in this area in any of the 120 samples analyzed. Therefore, when the arcuate eminence is abraded while using Fisch’s method, the posterior and anterior sides are safe areas for operation. The surgeon should start from these locations and gradually abrade the SSC elsewhere, so as to avoid direct injury to the SSC and reduce the risk of postoperative complications.

### The angle formed by the SSC and the internal auditory canal

The vector $\stackrel{`}{\text{F}\text{G}}$ was used to fit the IAC, and the vector $\stackrel{`}{\text{M}\text{B}}$ was used to fit the SSC. The angle (θ) formed by these two vectors ranged from 0–60°, as previously reported by Fisch. However, we also found that θ typically ranged from 10 to 30°. Therefore, if a fan with a top angle of 20° is removed on the basis of $\stackrel{`}{\text{M}\text{B}}$, the vector $\stackrel{`}{\text{F}\text{G}}$ can be found, which means that the IAC can be found. Therefore, in seeking to reduce the size of the artifactual lesion, there is still room for improvement in this step of Fisch’s method. However, since the vector $\stackrel{`}{\text{M}\text{B}}$ is not the “blue line” seen in clinical practice, additional studies are needed.

The posterolateral section of the middle cranial fossa is mainly composed of the petrous portion of the temporal bone, which is the main area operated upon during surgery on the lateral skull base. Due to the lack of clear anatomical signs provided by the middle skull base, in addition to the vulnerability of the cochlea, SSC, facial nerve, internal carotid artery, and other important structures under the thin skull base, the primary technical challenge is how to safely and accurately locate the IAC as it travels through the middle skull base. In this study, we used high-resolution CT reconstruction technology to measure the relationship between the IAC and anatomical reference points on the middle skull base in a patient population of Chinese ethnicity. The data presented and the conclusions drawn therefrom should allow surgeons to expose the IAC more safely and reliably during surgery, so as to avoid complications.

The main limitation of this study is that the anatomical positional relationship between the IAC and related structures of the middle cranial fossa was measured only in normal people, but not in patients with middle ear and/or mastoid disease. Genc et al. [17] and Karatag et al. [18] found that tympanitis and mastoiditis resulted in hypoplasia of the mastoid. Otitis media can also result in hypoplasia of the mastoid. Regardless of pathology, mastoid hypoplasia results in a low mastoid and brain plate. This suggests that otitis media and mastoiditis may also affect development of the lateral skull base. Although there is a lack of relevant research, whether the conclusions of this study can be applied to patients with middle ear and/or mastoid disease remains to be further verified. Additional factors that may affect the ability to extrapolate from the results presented here include the fact that the sample size of the study was small, and sampling errors may have affected the results. Research continues to expand on this issue in an attempt to refine the crucial relationship between the arcuate eminence and superior semicircular canal, with the latest data suggesting that the relationship is direct only when the arcuate eminence is flat, and is further dependent on the perilabyrinthine cells and cancellous bone around these two structures [19].

## Conclusions

In conclusion, the difficulty of Fisch’s method lies in finding the arcuate eminence. It is also difficult to determine whether the SSC lies below the arcuate eminence after the latter is removed. Some of the samples included in this study appeared to lack an arcuate eminence, which prevented the use of Fisch’s method. Fisch’s method sometimes oversimplifies the relationship between the arcuate eminence and the SSC, leading the surgeon to believe that the SSC was located directly below the arcuate eminence and resulting in intraoperative injury of the SSC. However, this study also showed that the angle typically ranged from 10 to 30 degrees. Fisch’s interpretation of the angle between the SCC and the IAC thus requires additional attention.

The authors concede that no clinical application of this research currently exists in practice. Our study focused on the existing Fisch classification in use and some of the deficiencies of the same, which if improved upon, could optimize clinical and surgical outcomes.

## Data Availability

The datasets generated and analyzed during the current study are not publicly available as none of the data types require upload to a public repository but are available from the corresponding author on reasonable request.

## Abbreviations

CT: computed tomography.
SSC: superior semicircular canal.
